# Phytochemical Profile, Biological Properties, and Food Applications of the Medicinal Plant *Syzygium cumini*

**DOI:** 10.3390/foods11030378

**Published:** 2022-01-28

**Authors:** Muhammad Qamar, Saeed Akhtar, Tariq Ismail, Muqeet Wahid, Malik Waseem Abbas, Mohammad S. Mubarak, Ye Yuan, Ross T. Barnard, Zyta M. Ziora, Tuba Esatbeyoglu

**Affiliations:** 1 Institute of Food Science and Nutrition, Bahauddin Zakariya University, Multan 60800, Pakistan; Muhammad.qamar44@gmail.com (M.Q.); saeedbzu@yahoo.com (S.A.); ammarbintariq@yahoo.com (T.I.); 2 Department of Food Technology, Engineering and Nutrition, Lund University, P.O. Box 188, SE-221 00 Lund, Sweden; 3 Department of Pharmacy, Bahauddin Zakariya University, Multan 60800, Pakistan; muqeetsoomro@msn.com; 4 Institute of Chemical Sciences, Bahauddin Zakariya University, Multan 60800, Pakistan; wasimchemist229@gmail.com; 5 Department of Chemistry, The University of Jordan, Amman 11942, Jordan; mmubarak@ju.edu.jo; 6 Institute for Molecular Bioscience, The University of Queensland, Brisbane, QLD 4072, Australia; ye.yuan1@uq.net.au (Y.Y.); Z.ZIORA@imb.uq.edu.au (Z.M.Z.); 7 School of Chemistry and Molecular Biosciences, The University of Queensland, Brisbane, QLD 4072, Australia; rossbarnard@uq.edu.au; 8 Institute of Food Science and Human Nutrition, Department of Food Development and Food Quality, Gottfried Wilhelm Leibniz University Hannover, Am Kleinen Felde 30, 30167 Hannover, Germany

**Keywords:** jamun, nutrition, antioxidant, inflammation, cancer, radioprotection, diabetes, hyperlipidemia, value addition, packaging

## Abstract

*Syzygium cumini*, locally known as Jamun in Asia, is a fruit-bearing crop belonging to the Myrtaceae family. This study aims to summarize the most recent literature related to botany, traditional applications, phytochemical ingredients, pharmacological activities, nutrition, and potential food applications of *S. cumini*. Traditionally, *S. cumini* has been utilized to combat diabetes and dysentery, and it is given to females with a history of abortions. Anatomical parts of *S. cumini* exhibit therapeutic potentials including antioxidant, anti-inflammatory, analgesic, antipyretic, antimalarial, anticancer, and antidiabetic activities attributed to the presence of various primary and secondary metabolites such as carbohydrates, proteins, amino acids, alkaloids, flavonoids (i.e., quercetin, myricetin, kaempferol), phenolic acids (gallic acid, caffeic acid, ellagic acid) and anthocyanins (delphinidin-3,5-*O*-diglucoside, petunidin-3,5-*O*-diglucoside, malvidin-3,5-*O*-diglucoside). Different fruit parts of *S. cumini* have been employed to enhance the nutritional and overall quality of jams, jellies, wines, and fermented products. Today, *S. cumini* is also used in edible films. So, we believe that *S. cumini*’s anatomical parts, extracts, and isolated compounds can be used in the food industry with applications in food packaging and as food additives. Future research should focus on the isolation and purification of compounds from *S. cumini* to treat various disorders. More importantly, clinical trials are required to develop low-cost medications with a low therapeutic index.

## 1. Botanical Description and Traditional Uses

**Figure 1:**
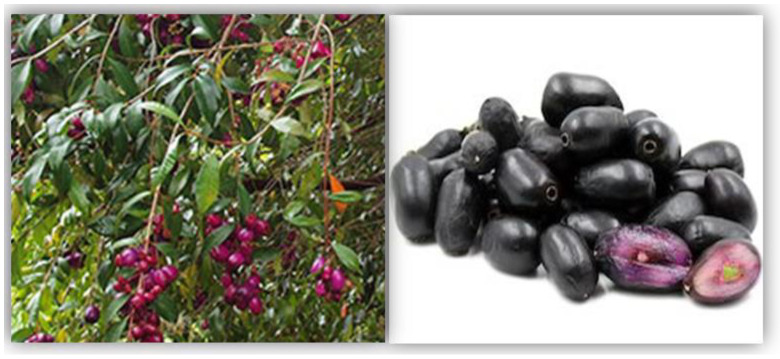
Leaves and fruits of *Syzygium Cumini*.

*Syzygium cumini* (L.) (synonyms: Eugenia jambolana, Syzygium jambolana, Eugenia cuminii), commonly known as jamun, jambul, jambolao, Java plum, Indian blackberry, and black plum, belongs to the family Myrtaceae. The fruit is native to South Asia, mainly Pakistan, India, Afghanistan, and Myanmar, and Pacific-Asia, including Indonesia, the Philippines, Hawaii, and Australia, and it is also cultivated in Florida and Kenya. During ripening, the fruit is greenish and at maturity pink to shining crimson (Figure 1). The harvesting period of the fruit jamun in Asia starts usually in the monsoon season (June to July) and lasts 30 to 40 days [1]. *S. cumini* fruits (1.5 to 3.5 cm) exhibit sweet flavor and mild astringency [2]. Bitterness can be reduced by pickling, adding some salt, and standing for a minimum of 1 h [3]. *S. cumini* fruits are eaten fresh, or as chutney, and jam. *S. cumini* juice is used for preparing summer drinks such as syrup, sherbet, and squash. The squeezed fruits are normally heated for 10 min and mixed with water, sugar, citric acid, and sodium benzoate for preservation [2].

*S. cumini* has been traditionally used as a medicinal plant. Different parts of the plant (for example bark, leaves, seeds, and fruit) have been employed in the treatment of various diseases. *S. cumini* fruit juice has been utilized, orally, to treat gastric complaints, diabetes, and dysentery [4]. *S. cumini* seeds have been applied externally to treat ulcers and sores, and powdered seeds with sugar have been given orally to combat dysentery [5]. Powdered seeds have been reported to be effective against diabetes [6]. *S. cumini* leaves were cooked in water (concentration of 2.5 g/L) and drunk daily, where 1 L has been reported to be effective against diabetes [7]. The juice of leaves has been used as an antidote in opium poisoning, and an oral intake of leaves for 2–3 days has been reported to be effective in reducing jaundice in adults and children [8]. Traditionally, *S. cumini* leaves juice along with mango leaves and myrobalan fruit administered with honey and goat milk has been used also to combat dysentery [9], whereas bark decoction of *S. cumini* with water has been used to treat diabetes [10], dysentery, to increase appetite, to achieve sedation, and to relieve headache when taken orally [4]. Bark decoction has been given to females with recurrent miscarriages [5]. *S. cumini* bark juice with buttermilk has been reported to treat constipation, whereas an intake in the morning has been claimed to stop blood discharge in feces [11].

## 2. Phytochemical Profile

*S. cumini* fruits contain high amounts of vitamins, minerals, and fiber. They are low in calories and fat [1]. Phytochemicals of *S. cumini* including carbohydrates, proteins, fats, fiber, minerals, and vitamins are listed in Table 1.

Analysis of *S. cumini* fruits yielded moisture, protein, sugar, and ash contents as 80.8, 0.81, 12.7 and 0.70% on a fresh weight basis, respectively [22], which is in agreement with more recent results: moisture (79.2%), protein (0.65%), sugar (7.88%), ash (1.03%), and fat (0.18%) contents on a fresh weight basis [3]. Octadecane (16.9%), nonacosane (9.9%), and triacontane (9.3%) are dominant constituents of the leaf oil, whereas octacosane (7.4%), hepatcosane (4.8%), hexadecanoic acid (4.2%), and eicosane (4.02%) are also present [21,22,23]. Additionally, *S. cumini* seeds have fatty oils such as oleic acid (32.2%), myristic acid (31.7%), and linoleic acid (16.1%) as their main constituents. However, stearic acid (6.50%), palmitic acid (4.70%), lauric acid (2.80%), vernolic acid (3.00%), sterculic acid (1.80%), and malic acid (1.20%) were detected in small quantities [24]. The seed oil mainly comprised of 1-chlorooctadecane followed by tetracontane, decahydro-8a-ethyl-1,1,4a,6-tetramethylnapahthalene, 4-(2-2-dimethyl-6-6-methylene-cyclohexene) butanol, octadecane, octacosane, heptacosane, and eicosane in the range of 33.2%, 9.24%, 8.02%, 5.29, 5.15%, 3.97%, 1.72%, and 1.71%, respectively [23]. On the other hand, leaves contain minerals such as sodium, potassium, calcium, zinc, iron, magnesium, copper, manganese, lead, and chromium [3]. Recently, the foliar application of zinc and boron was reported to exert profound effects on fruit weight (10.29 to 12.88 g), seed weight (1.68 to 2.55 g), and fruit length (19.55 to 25.88 mm). Other physicochemical parameters including total soluble solids and titratable acidity showed no change upon foliar treatment with zinc or boron, but it slightly influenced the reducing sugar content of *S. cumini* fruit; i.e., it increased from 6.33 to 6.64% [25].

Recent databases suggest that different plant parts including skin and pulps, essential oils, seeds, flowers, barks, and leaves have different and characteristic compositions (Table 2) [26,27,28,29,30,31]. 

The purple shade of *S. cumini* fruit is due to the presence of anthocyanins, whereas its astringent flavor is imparted by a high concentration of tannins [15]. Furthermore, 3,5-diglucosides of malvidin, delphinidin, and petunidin were identified in *S. cumini* peels [26,27]. Bioactive components such as phenolic acids, gallic acid, ellagic acid, carotenoids, flavonoids, myricetin, and their derivatives were identified in fruit pulps [28,29]. About 30 compounds such as terpinyl isovalerate, dihydrocarvyl acetate, and geranyl butyrate contribute to the flavor of the purple fruits [30]. Moreover, a qualitative investigation of *S. cumini* seeds unveiled the presence of gallic acid, ellagic acid, corilagin, jambosine, quercetin, and *β*-sitosterol [40]. Recently, the essential oil of *S. cumini* leaves was analyzed by GC-MS, wherein τ-cadinol and τ-muurolol were found in high amounts as 21.4% and 12.4% of the total oil fraction, respectively [31].

## 3. Pharmacological Potential of *S. cumini*

*S. cumini* has been used in various ancient medicinal systems such as Siddha, Tibetan, Unani, Sri Lankan, and Ayurveda. It was employed in the aforementioned systems with a view to curing diarrhea, menstrual disorders, obesity, hemorrhage, and vaginal discharge [41]. Parts of *S. cumini* including fruit, seed, bark, leaves, pulp, and skin are known for their antioxidant [42], anti-inflammatory [43], anticancer [44], and antidiabetic activities [7]. Data are available on animals and different in vitro models to support their hepatoprotective [45], cardioprotective [46], chemopreventive potential [47], and antipyretic properties [48]. Some investigations have revealed activity against diabetes [49], obesity [50], inflammation [48], and bacterial infections [51]. Below are details about the documented pharmacological activities of *S. cumini*.

### 3.1. Antioxidant Activity

An imbalance between endogenous antioxidant defense and reactive oxygen species is the main reason for oxidative stress, and it has been suggested as a principal cause of the eventual inception of ailments. Antioxidants are considered to be key ingredients imparting health and protecting against various infections and degenerative diseases through radical scavenging capacity [52]. The antioxidant potential of *S. cumini* extracts has been explored by various researchers by employing a variety of in vitro assays including nitric oxide (NO), 2,2-diphenyl-1-picrylhydrazyl (DPPH), ferric-reducing antioxidant power (FRAP), 2,2′-azino-bis (3-ethylbenzothiazoline-6-sulfonic acid) (ABTS), and oxygen radical absorbance capacity (ORAC) [36,53,54,55,56,57,58]. Within this context, investigations by Veigas et al. (2007) [26] revealed that anthocyanins extracted from *S. cumini* fruit peels using acidified methanol cause noteworthy protection against iron-induced lipid peroxidation. Similarly, *S. cumini* peel extract was reported to exert more potential (90.6%) against DPPH than pulp (82.5%) and seed (85.2%) extracts due to the presence of high phenolic (4812–5990 mg gallic acid equivalent (GAE)/100 g) contents on a dry weight (dw) basis [59]. Likewise, a considerable amount of tannins isolated from *S. cumini* peel using 70% aqueous acetone showed significant radical scavenging activities using the DPPH and FRAP assays [42]. Identically, freeze-dried extract of *S. cumini* pulp showed values of 670 mM Trolox equivalent (TE)/100 g (DPPH), 820 mM TE/100 g (ABTS), and 750 mM TE/100 g dw (FRAP) [60].

Vasi and Austin (2009) [56] reported that 50% aqueous ethanol *S. cumini* seed extract causes a six-fold greater scavenging activity against ABTS (98.9%), NO (96.8%), FRAP (94.4%), and DPPH (92.3%) as compared to the standard Trolox. Furthermore, a significant alteration was observed against the elevated level of peroxides and reduced level of antioxidant status including catalase, superoxide dismutase (SOD), glutathione (GSH), and ascorbic acid after oral administration of *S. cumini* 90% aqueous methanol seed extract in rats [61]. Water *S. cumini* seed extract showed better ORAC values (338 mM TE/100 g) than pulp extract (144.5 mM TE/100 g) as well as reducing oxidative stress [47,58]. Moreover, *S. cumini* methanol leaf extract contained significant amounts of total phenolics and flavonoids, with good antioxidant status, suggesting the potential of *S. cumini* to treat various human disorders [62].

Recent investigations have suggested that ethanol extracts of *S. cumini* parts (stem, leaf, seed) possess the protruding potential to reverse oxidation mechanism as compared to water extracts. In addition, the ethanol extracts of stem, leaf, and seed exhibited radical scavenging ability as 33%, 68%, and 98%, respectively in the DPPH assay [56]. Findings by Saeed et al. revealed that when evaluated for radical scavenging capacity, four different cultivars of *S. cumini* displayed more than 90% reduction against DPPH free radicals, which is attributed to the presence of large amounts of ascorbic acid, total phenolics, and anthocyanins [36]. In addition, the methanol leaf extract of *S. cumini* exhibited antioxidant activity at 1314 mg ascorbic acid equivalent (AAE)/100 g in the FRAP assay, whereas dichloromethane extract showed weaker activity at 122 mg AAE/100 g dw [63]. Furthermore, the ethanol leaf extract of *S. cumini* at 20 g/kg of body weight played a preventative role due to its antioxidant potential against gastric ulceration in rat stomach [64]. Similarly, the *S. cumini* bark extract can scavenge free radicals owing to the presence of a variety of bioactive compounds [65]. 

On the other hand, research findings showed that the *S. cumini* methanol leaf extract contains 369.75 mg GAE/g and 75.8 mg rutin equivalent (RE)/g total phenolic and flavonoid contents, respectively and exhibits notable inhibition activity with an IC_50_ of 133 μg/mL against stable free radicals i.e., DPPH comparable to the standard ascorbic acid (IC_50_ of 122.4 μg/mL) [66]. Moreover, three anthocyanins—delphinidin-3,5-*O*-diglucoside, petunidin 3,5-*O*-diglucoside, and malvidin-3,5-*O*-diglucoside—were isolated from *S. cumini* pulp using high-speed counter current chromatography; these compounds exhibited strong radical-scavenging abilities [67]. *S. cumini* leaf extract protected against paraquat-induced toxicity in *Saccharomyces cerevisiae* strains deficient in SOD owing to higher antioxidant activity [68]. Sequentially extracted ethyl acetate and *n*-butanol fractions of *S. cumini* leaves delineated notable radical-scavenging activity in the DPPH assay with IC_50_ 15.7 and 23.5 μg/mL, 1155 and 1178 μmol TE/g in the FRAP assay, and 1225 and 1314 μmol TE/g in the ORAC assay [29]. In addition, the *S. cumini* methanol fruit extract exhibited strong antioxidant properties in the DPPH (IC_50_ 81.4 μg/mL), FRAP (46.0 mmol Fe/g), and H_2_O_2_ (76% inhibition) assays as compared to 50% aqueous methanol and dichloromethane extracts [37]. Recently, Santos et al. (2021) [69] found that *S. cumini* water leaf extract is potent in averting the excessive generation of reactive oxygen/nitrogen species, macrophages viability loss, and foam cells formation that is caused by LDL oxidation.

### 3.2. Anti-Inflammatory Potential 

**Scheme 1:**
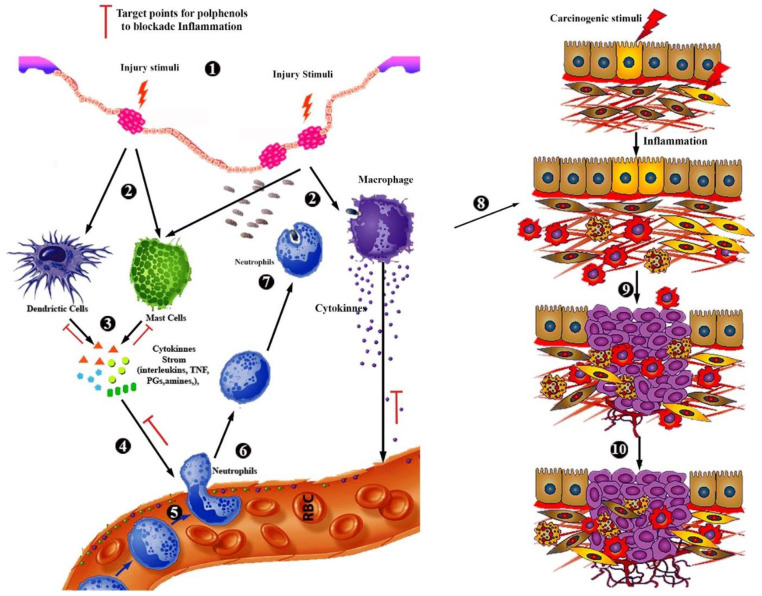
Mechanism of inflammation leading to carcinogenicity (signed by the authors Saeed Akhtar and Muqeet Wahid using Adobe). 1. Carcinogenic or injury stimuli cause the injury on the epithelium layer of tissue. 2. Activation of dendritic, mast cells, and macrophages present under epithelium layer in between parenchymal cells of the tissue. 3. Dendritic, mast cells, and macrophages release the proinflammatory cytokines (TNF, interleukins, PGs, amines, amines, etc.). 4. Cytokines act on blood vessels for vasoconstriction to migrate platelets and neutrophils from blood vessels to the site of inflammation. 5. Neutrophils roll and 6. Migrate to the site of inflammation. 7. Neutrophils engulf the microbes and necrotic cells. 8. Chronic inflammation may lead to the mutagenicity and proliferation of cells 9. Inflammation and mutagenicity lead to the uncontrolled proliferation of cells with hyperplasia and dysplasia. 10. Angiogenesis with uncontrolled proliferation of cells, which leads to tumor formation in tissue.

Excess production of free radicals from activated inflammatory leukocytes, especially under conditions of chronic inflammation, may have an important role in various pathologies. Several reports have suggested that diseases associated with inflammation may be ameliorated by polyphenols (Scheme 1).

In this regard, *S. cumini* is a well-reputed medicinal plant with anti-inflammatory potential. The *S. cumini* water fruit extract exhibited considerable anti-inflammatory activity of 68.9%, while the standard diclofenac holds an anti-inflammatory potential of 75.1% [43]. Similarly, a flavonoid-rich fraction of *S. cumini* fruits is reported to palliate inflammatory reactions in human lymphocytes, neutrophils, and monocytes against the hepatitis B vaccine [70]. In addition, ethanol, methanol, ethyl acetate, and water *S. cumini* seeds extracts are effective against carrageenan-induced paw edema in Wistar rats [71]. On the other hand, the *S. cumini* chloroform seed extract caused suppression of protein exudates, dye leakage during peritoneal inflammation, and migration of leukocytes along with inhibition of acute and sub-acute inflammation in rats [72]. Likewise, the water seed extract of *S. cumini* reduced inflammation through action on human neutrophils [73].

In a recent study, the water extract of *S. cumini* seeds alleviated inflammation by causing the suppression of ectonucleotidase, acetylcholinesterase, adenosine deaminase, and dipeptidyl peptidase IV, and inhibited NO activities [74]. Similarly, methanol seed extract of *S. cumini* displayed greater anti-inflammatory potential compared to fruit juice in hemagglutination inhibition and membrane stabilization assays; this is attributed to the high polyphenolic content present in the methanolic fraction [75]. Moreover, *S. cumini* leaf water extracts alleviated indomethacin-induced inflammation by suppressing cyclooxygenase (COX), inducible nitric oxide synthase (iNOS), and tumor necrosis factor-alpha (TNF-α) enzymes [76]. Recently, research findings revealed that *S. cumini* seeds alter the inflammatory responses induced by lipopolysaccharide (LPS) by suppressing jun N terminus kinase (JNK), extracellular signal-regulated kinase (ERK), and p38 arbitrating nuclear factor-κB (NF-κb) pathways as well as down-regulation of iNOS and COX-2 [77]. In addition, food fortification with *S. cumini* seeds protected rat kidneys and liver by attenuating inflammatory cell infiltration, collagen secretion, deposition of extracellular matrix, and overload of iron, which is attributed to the presence of antioxidants [78].

Studies involving animals showed that the ethanol stem bark extract of *S. cumini* exerts significant effects against inflammation induced by carrageenan, kaolin, and formaldehyde in rats with no gastric lesions [48]. Additionally, it was reported that the ethanolic bark extract of *S. cumini* exhibited inhibition against prostaglandin E2 (PGE2), 5-hydroxytryptamine (5-HT), and histamine-induced paw edema in rats, but was ineffective against bradykinins-induced inflammation [79]. Similarly, the ethanol root extract of *S. cumini* showed anti-inflammatory potential in vitro by reducing interleukin 6 (IL-6) in RAW 264.7 macrophages [80].

In addition, the tannin-free ethyl acetate fraction of *S. cumini* leaves possessed a concentration-dependent reduction of the polymer C48/80-induced paw edema even at doses as low as 0.01 mg/kg [81]. In the same manner, *S. cumini* leaf ethyl acetate and methanol extracts showed an inhibitory effect against carrageenan-induced inflammation at doses of 200 and 400 mg/kg [82]. Other researchers showed that the *S. cumini* leaf extract exerts anti-inflammatory effects against acute and chronic inflammation in albino rats [83]. Furthermore, published research indicated that the acetone extract of *S. cumini* leaves possesses both anti-hyperglycemic and anti-inflammatory activities [84]. Similarly, the methanol leaf extract of *S. cumini* and its subsequent fractions obtained using the Kupchan partitioning method exhibited a potent action against acetic acid-induced writhing in a dose-dependent manner [85].

Another study revealed that in both acute (carrageenan, serotonin, histamine) and chronic (cotton pallet rat granuloma) models of inflammation, *S. cumini* leaf methanol extract exhibits potent activity in a concentration-dependent manner [86]. Additionally, *S. cumini* tannin-rich fraction reduced inflammation by 99.5% in the heat-induced protein denaturation assay at 100 μg/mL better than the commercially available anti-inflammatory drug aspirin that caused inhibition of 89.3% at the same concentration. A similar trend has been observed in the human red blood cell membrane (HRBC) stabilization assay where *S. cumini* tannin-rich fraction exhibited 82.9% protection of HRBC membrane at a dose of 1 mg/mL while the standard diclofenac caused protection of 70.4% as compared to the control i.e., distilled water [87].

Moreover, essential oils from *S. cumini* reduced chronic granulomatous inflammation in BALB/c mice and inhibited the migration of rat eosinophils, thus showing anti-inflammatory activity [88,89]. In addition, *S. cumini* methanol leaf extract exerted analgesic effects in rabbits as evident from decline in writhing (12.2 control vs. 3.7 treated) and anti-inflammatory response, i.e., 64.1% inhibition [66]. Furthermore, malvidin-3,5-*O*-diglucoside, an anthocyanin isolated from *S. cumini* pulp, inhibited the release of nitric oxide and pro-inflammatory mediators including IL-6, IL-1β, and TNF-α in LPS-induced RAW264.7 macrophages [67]. Similarly, methanol fruit extract of *S. cumini* exhibited significant in vitro (heat-induced hemolysis, albumin denaturation, bovine serum albumin denaturation) and in vivo (carrageenan-induced paw edema, formaldehyde-induced paw edema, PGE2-induced paw edema) anti-inflammatory activities, which were considerably higher than 50% aqueous methanol and dichloromethane extracts [37]. In Table 3, the anti-inflammatory activities of different parts of *S. cumini* are shown.

### 3.3. Anticancer Potential

Cancer is among the most common life-threatening maladies and is a prime cause of death worldwide. According to Global Cancer Statistics, Asia including Southern Asia (India, Sri Lanka), South-Eastern Asia (Myanmar, Philippines, Indonesia, Cambodia, Vietnam), and Eastern Asia (China, Japan, Republic of Korea, Mongolia) is leading with its worldwide share of cancer deaths (57.3%). Approximately one-half of all new cancer cases and above one-half of the overall cancer deaths worldwide were reported from Asia, while breast cancer and lung cancer are the commonly diagnosed and leading cause of death in females (11.6%) and males (18.4%), respectively [90]. Various therapeutic drugs in Western medicine have been used to treat cancer-related diseases. Some of these have serious toxicity and side effects. Chemotherapy is a widely used technique for cancer treatment, but its adverse effects on normal cells cannot be neglected [91], so there is a need to discover chemical compounds that are effective and have low off-target toxicity (that is, they possess a high therapeutic index). In this respect, the application of phytochemicals for cancer chemoprevention could be a promising approach to reduce cancer prevalence. Medicinal plants are a “herbarium” of bioactive compounds and could be used as chemopreventive agents [92].

*S. cumini* crude and methanol extracts were evaluated for cytotoxic activity against cultured human cancer cells, SiHa (carcinoma of uterus) and HeLa (cervical cancer). Results revealed a dose-dependent cell death against both cancer cell lines. Extracts showed more pronounced activity against HeLa than SiHa cell lines. Moreover, the 50% aqueous methanol fraction was reported to induce greater apoptosis in HeLa compared to SiHa cells [93]. A study conducted by Li et al. (2009) [27] investigated the anti-proliferative and pro-apoptotic effects of a standardized *S. cumini* fruit extract. Standardized anthocyanins fraction was reported to possess a significant effect against MCF-7 with an IC_50_ of 27 μg/mL, while an IC_50_ of 40 μg/mL was reported against MDA-MB-231 breast cancer cell lines. In contrast, the standardized *S. cumini* fruit extract was reported to cause no cell death and apoptosis against a normal breast cell line MCF-10A with an IC_50_ of >100 μg/mL. Furthermore, *S. cumini* seed ethanol extract exhibited an anticancer effect with IC_50_ values of 49, 110, 140, and 165 μg/mL against ovarian (A2780), breast (MCF-7), prostate (PC-3), and lung (H460) cancer cell lines, respectively. The ovarian cancer line was found to be most susceptible to seed extract, while non-significant results were obtained against breast, prostate, and lung cancer cell lines [51]. Furthermore, methanol and ethyl acetate fractions of *S. cumini* seeds were reported to cause concentration-dependent activity against MCF-7. In addition, the ethyl acetate extract was found to be more promising compared to a methanol extract, and a similar trend was observed for DNA fragmentation, which is considered as an apoptosis indicator [94]. In Table 4, the anticancer properties of *S. cumini* are summarized.

Charepalli et al. (2016) [97] reported the anticancer activity of anthocyanins isolated from *S. cumini* fruits using acidified methanol. The results revealed that *S. cumini* fruit triggered cytotoxic effects against HCT-116 colon cancer cells in a dose-dependent mode. Additionally, *S. cumini* fruit anthocyanin fractions provoked DNA fragmentation and caused apoptosis in colon cancer cell lines and colon cancer stem cells, as evaluated through caspase 3/7 and terminal deoxynucleotidyl transferase-mediated dUTP nick end labeling (TUNEL) assays. Similarly, *S. cumini* methanol fruit extract elevated cytotoxicity and the suppression of cell proliferation in H460 lung cancer cells (IC_50_ of 35.2 µg/mL) in a dose-dependent manner [100]. At the same time, Aqil et al. [44] reported that *S. cumini* holds the appreciable potential to balance estrogen-mediated alterations in mammary cell-proliferation, estrogen receptor-alpha (ER-α), cyclin D1, and miRNAs, and that the modulation of these biomarkers correlated with a reduction in mammary carcinogenicity. Recently, a fraction of a *S. cumini* ethanol leaf extract, obtained from column chromatography, caused significant inhibition (69%) against the T47D breast cancer cell line [49]. Consistent with these observations, key findings of Ezhilarasan et al. (2019) [95] showed that *S. cumini* methanol fruit extract causes an inhibition of oral squamous cell carcinomas cells (OSCC). In addition, the ethanolic fraction of *S. cumini* fruit exhibited notable anti-leukemia activities, which is a direct correlation among polyphenolic contents, antioxidant status, and anticancer potential [96]. Furthermore, findings of Goyal et al. (2010) [101] revealed that pre, post, and pre–post oral administration of *S. cumini* extract induces an appreciable reduction in chemically induced tumor incidence, tumor burden, and a cumulative number of gastric carcinomas. The *S. cumini* extract also inhibits the reduction of phase II enzymes, which exhibit detoxification properties and lipid peroxidation [101]. Moreover, the *S. cumini* methanol leaf extract indicated a concentration-dependent cytoprotective activity against H_2_O_2_-treated bone marrow mesenchymal stem cells (BM-MSCs) [66]. Recently, *S. cumini* ethanol extract reported to inhibit the proliferation of HT-29 cells further confirmed by a DNA damage assay wherein DNA lost its integrity and went through apoptosis. The wound healing also proposed the lower change of metastasis when treated with *S. cumini* ethanol extract [98]. Recent findings showed that seed and leaf methanol extracts of *S. cumini* inhibit the growth of colon cancer cell line in a dose-dependent manner with IC_50_ values of 1.24 and 1.42 µg/mL, whereas the IC_50_ of the standard drug doxorubicin was 1.14 µg/mL [99]. 

### 3.4. Radioprotection 

Phenolic compounds are known to be radioprotective with high biopotency as UV-A and UV-B blockers. Jagetia et al. (2003) [102] revealed that the application of dichloromethane and methanol leaf extracts of *S. cumini* on human peripheral blood lymphocytes before irradiation (3 Gy) results in significant protection of DNA. Additionally, protection against radiation-induced mortality and sickness in mice was also observed for the same extracts at 30 mg/kg [102]. Similarly, pretreatment with dichloromethane and methanol leaf extracts of *S. cumini* induced significant protection in mouse intestine against γ-radiation by increasing the number of regenerating crypts and rise in villus height, which is accompanied by a reduction in goblet and dead cells, which caused an increase in the life span of irradiated mice [103]. Likewise, dichloromethane and methanol leaf extracts of *S. cumini* caused inhibition against radiation-induced micronuclei generation, representing the potential against radiation-induced DNA damage [104]. Furthermore, the application of dichloromethane and methanol leaf extracts of *S. cumini* before irradiation caused an increase in GSH, catalase, and SOD levels. A dose of 50 mg/kg reduced lipid peroxidation in mouse liver [105]. Similar investigations revealed that pretreatment of mice with *S. cumini* of 50% aqueous methanol seed fraction against a lethal dose of 10 Gy alters the radiation-induced sickness and mortality. Furthermore, research findings indicated that the best protection against irradiation was at 80 mg/kg when applied intraperitoneally. The survival rate observed for the intraperitoneal route was found to be almost double (50%) compared with the oral route (22%) and 0% in the radiation alone [106]. All of these observations signify that *S. cumini* holds great capabilities to protect against radiation-induced sickness, intestinal, and DNA damage by increasing antioxidants that counter radiation-induced free radicals. *S. cumini* leaf water extract also contributed toward radioprotection by suppressing inflammatory cytokines such as TNF-α, NF-κB, iNOS, and COX enzymes [76]. Recently, a significant increase in GSH levels and wound healing were observed after oral and topical administrations of *S. cumini* seeds ethanol extract along with topical laser therapy [107].

### 3.5. Hyperlipidemia and Cardioprotective Activity

An abnormal rise in blood lipid contents is known as hyperlipidemia. Any deviation in the lipid profile results in several heart issues inclusive myocardial infarction, cardiovascular diseases (CVD), stroke, and atherosclerosis [108]. Medicinal plants gained attention for the treatment of lipidemia and associated ailments due to the side effects of synthetic drugs [109]. Along this line, the *S. cumini* seeds flavonoid-rich fraction was evaluated for antilipidemic potential; results showed a reduction in low-density lipoproteins (LDL) levels and elevation of high-density lipoproteins (HDL) levels in rats [110]. Similarly, the fraction reduced the serum lipid levels [111]. Similarly, *S. cumini* seed water extract reduced triglycerides and LDL levels, whereas elevation of HDL levels was observed in alloxan-treated mice. Research findings also showed cardioprotective effects of *S. cumini* fruit ethanol extract [112]. Cardiovascular diseases are one of the important causes of death in industrialized nations, and hyperlipidemia is one of the main causes of cardiovascular ailments [109]. In this respect, work by Nahid et al. (2017) [113] revealed that methanol seed extract of *S. cumini* exhibits cardioprotective activity in isoproterenol-induced myocardial infarction in rats. Oral feeding for 30 days resulted in a concentration-dependent protection against the myocardial infarction. Application of the same extract caused alteration against cardiac and liver damage in diabetic rats. The *S. cumini* leaf methanol extract also possesses significant anticoagulant activity with a prothrombin time of 28.3 s vs. 15.8 s of control [66].

### 3.6. Antidiabetic Potential

In the last three decades, a considerable increase in type 2 diabetes cases, especially from developing countries where about 80% of the people have diabetes, was reported, and the rise of type 2 diabetes in South Asia is estimated to be more than 150% between 2000 and 2035 [114]. Antidiabetic properties of various parts of *S. cumini* including pulp, seed, bark, and stem have been investigated [7]. In this respect, Achrekar et al. (1991) [115] reported good antidiabetic activity of a water extract of *S. cumini* pulps; it caused a significant reduction in blood glucose level in streptozotocin-induced diabetic rats. The hypoglycemic index depended on the level of injected dose and mode of administration. Similarly, researchers showed that an excellent hypoglycemic index is achieved at about 100 to 200 mg/body weight with the intraperitoneal injection route [116]. In addition, results of rat modeling for evaluating hypoglycemic activity supported its antidiabetic potential and effectiveness against alloxan-induced diabetes [117]. Moreover, research findings indicated that the oral administration of *S. cumini* water and methanol extracts into rabbits at 200 and 300 mg/kg body weight resulted in almost the same hypoglycemic effect achieved with the standard drug tolbutamide at 100 mg/kg body weight [118].

Similarly, *S. cumini* seeds exhibit hypoglycemic activity as determined by the reduction of blood glucose levels in alloxan-induced diabetic rabbits. Results showed that ethanol extract of seeds at 100 mg/kg body weight caused a substantial reduction of fasting blood glucose levels. Histopathological analysis of various organs supported the hypoglycemic activity of *S. cumini* seed extract [117,118]. *S. cumini* seed powder [119] or extract (ethanol, methanol, ethyl acetate, and water) administered via different routes (oral, intraperitoneal) have the potential to control diabetes [117,120,121,122,123,124]. In a clinical trial, Sahana et al. (2010) [125] reported the effectiveness of *S. cumini* seed dosage by the oral route against diabetes (type 2) with no toxic effects on human subjects. Moreover, research findings supported the antidiabetic activity of *S. cumini* seeds due to the reduction of carbohydrate-hydrolyzing enzymes by the carotenoid luteolin, which binds at the α-amylase site and acts as an inhibitor of carbohydrate breakdown similar to acarbose [126]. This mechanism of action and results aligned with previous research studies on antidiabetic potential conducted by Karthic et al. (2008) [127] and Ponnusamy et al. (2011) [128]. These researchers suggested that seed extracts control amylase, pancreatic amylase, glucoamylase, and other starch-hydrolyzing enzymes.

When streptozotocin-induced diabetic rats were treated with ethanol extract of *S. cumini* seeds, blood sugar levels were significantly reduced [129,130], whilst a methanol extract has been reported to control serum glucose level in alloxan-induced diabetic rats [131]. Moreover, *S. cumini* seed lyophilized powder caused a significant reduction in sugar levels in mice and rats [123,124]. Similarly, water extract of *S. cumini* seeds also controlled blood glucose levels in alloxan-induced diabetic mice [132]. Interestingly, the water-soluble seed powder of *S. cumini* with gummy fiber was found to control diabetes, whereas other preparations without fibrous matter failed to control diabetes in alloxan-induced diabetic rats [133]. Not only *S. cumini* seed extracts but also its individual bioactive compounds, such as mycaminose, play a significant role in controlling diabetes induced by streptozotocin in rats [134]. In this respect, ethanol extract of *S. cumini* seed kernel significantly reduced GSH levels in the liver and kidney of streptozotocin-induced diabetic rats [135,136].

In addition to the edible portions, including fruit and seed powder, leaves of *S. cumini* are potent in lowering glucose levels in both the blood and serum of experimental diabetic animals [137]. Sharafeldin et al. (2015) [138] extended their research by using extracts of *S. cumini* fruit and *Cinnamon zeylanicum* stem bark in combination and generated better results compared to their isolated effects. Furthermore, *S. cumini* seeds water extract at 400 mg/kg was more effective in controlling blood glucose levels in type II diabetes and in controlling peroxisome proliferator-activated receptor (PPARγ and PPARα) proteins of the liver [139]. *S. cumini* extract prepared from its stem bark was also found effective in controlling blood sugar levels against diabetic rats [140]. Accordingly, *S. cumini* seed ethanol extract was reported to reduce blood glucose levels [107]. Recently, sequentially extracted fractions of *S. cumini* leaves, i.e., dichloromethane, ethyl acetate, and *n*-butanol, were reported to cause 100% inhibition of α-amylase, wherein these fractions evinced 50% inhibition against α-glucosidase [29]. Recently, *S. cumini* seed extract (200 mg/kg) in combination with the standard drug metformin showed notable anti-hyperglycemic effects in streptozotocin-induced diabetic rats [141].

### 3.7. Gastroprotective, Antidiarrheal, and Antimicrobial Activity

Gastric ulcer is the most commonly diagnosed illness of the human digestive system [142]. Previous investigations revealed that the ethanol extract of *S. cumini* seeds reduces streptozotocin- and ethanol-induced peptic ulcers [129,130]. In addition, research findings showed that tannins from *S. cumini* offer excellent protection from hydrochloric acid- and ethanol-induced gastric ulceration by minimizing the gastric mucosal damage [64]. *S. cumini* seeds mixed with jaggery (non-centrifugal cane sugar) were reported to impart relief from diarrhea and dysentery. Likewise, tannins present in *S. cumini* fruits are well-known for their anti-diarrheal potential [143]. Moreover, *S. cumini* seed and flower with silver nanoparticles (AgNPs) exhibited notable antimicrobial potential when tested at concentrations between 31.2 and 2000 μg/mL against several bacterial and fungal species such as *A. naeslundii, C. albicans, F. nucleatum, S. aureus, S. epidermidis, S. mutans, S. oralis,* and *V.dispar* comparable with the activity of crude extracts tested at concentrations between 648 and 5188 μg/mL [144].

## 4. Value-Added Food Products and Food Packaging Material 

*S. cumini* fruit powder was supplemented with gum arabic, which was considered to be an appropriate carrier of the food components, because it retained the maximum functional attributes i.e., total flavonoid content, total phenolic content, and total anthocyanin content, in contrast to maltodextrin and combination of maltodextrin/gum arabic [145]. Similarly, *S. cumini* pomace was added to ice cream at doses of 1, 2, 3, and 4% and evaluated the change in physicochemical features of ice cream. The addition of *S. cumini* pomace induced a considerable increase in titratable acidity, fiber contents, ash, hardness, and total soluble solids, wherein a notable decline was observed for pH, melting rate, and fat contents. Moreover, the best sensory attributes were obtained when ice cream was treated with 3% of *S. cumini* pomace [146]. Another study conducted by Talukder et al. (2020) [147] made use of the unique feature of anthocyanins (change in color with the change in pH) and applied *S. cumini* anthocyanins as a quality indicator for meat products. Anthocyanin-rich skin extract of *S. cumini* was immobilized on filter paper strips. The strips, when attached inside a packet of chicken patties, changed color from violet to yellow as the pH varied, which was due to the reaction with volatile basic compounds produced from the meat stored at 4 °C. Observations suggested that the change in the color pattern is directly associated with the quality traits of chicken patties. For example, during a 21-day storage trial, numerous changes in the quality features of chicken patties were observed, including a decrease in pH from 6.22 to 6.04, increased level of nitrogen and ammonia, change in color, decrease in sensory attributes, and an increase in microbial count. Consequently, it has been anticipated that *S. cumini* skin extract filter paper can offer a suitable, non-toxic, visual means to check the quality of meat products during freezing and storage.

Kapoor et al. (2021) [148] prepared antioxidant-rich snacks by supplementing the rice flour with hot air-dried and freeze-dried *S. cumini* powder as 5, 10, 15, and 20% that substantially influenced the quality parameters of snacks. A considerable decrease in water absorption index and the expansion ratio was observed, along with an increase in water solubility, bulk density (g/cm^3^), and hardness. The best sensory attributes were found for 10% *S. cumini* supplemented snacks (freeze-dried and hot air-dried). Moreover, freeze-dried *S. cumini* powder supplemented snacks displayed 9.52% more antioxidant potential in comparison to hot air-dried *S. cumini* supplemented snacks. In addition, the total phenolic contents were 89.7% and 80.4% in supplemented snacks comprising freeze-dried and hot air-dried *S. cumini* powder, respectively, at a 10% substitution level.

On the other hand, wheat pasta made with 30% *S. cumini* pulp was found to be the best in terms of overall acceptability based on sensory and phytochemical parameters [149]. In this case, the addition of *S. cumini* pulp enhanced the free radical-scavenging potential (5.76 to 10.2%), *β*-carotene level (1336 to 7624 µg/100 g), total phenolics (111 to 176 mg GAE/100 g), dietary fiber (7.08 to 16.6%), and ash content (0.59 to 2.96%). Gruel or cooking loss was more but within an acceptable range i.e., below 10% with the addition of *S. cumini* pulp. Furthermore, an inverse proportionality was observed between pulp concentration and cooking time, lightness, and yellowness while redness increased with the incremental addition of *S. cumini* pulp.

Recently, active and pH-sensitive edible films were developed by amalgamating the *S. cumini* skin extract into methylcellulose films. The addition of *S. cumini* skin extract elevated the mechanical and barrier properties of methylcellulose films. Moreover, films added with *S. cumini* skin extracts exhibited alike antioxidant potential compared to pure extracts. Analysis revealed that the resulting active intelligent films are biodegraded in seawater in two days and soil in 15 days. The developed films can offer an enhanced shelf-life, preserve product freshness, lessen atmospheric pollution, and can be applied in the meat and aquatic food industry where lipid oxidation and change in pH can spoil the food [150]. Similarly, Merz et al. (2020) [151] developed and characterized colorimetric indicator films from polyvinyl alcohol, chitosan, and most importantly anthocyanins from *S. cumini* fruit. Results revealed that the addition of anthocyanins positively influences the thickness and optical attributes of films. The developed films comprising anthocyanins presented discernable variations from red to blue color when employed to evaluate the freshness of shrimps at numerous temperatures ranging from −20 to 20 °C, which can be considered as alternative option in the food packaging industry. Kasai et al. (2018) [152] made films adopting different combinations of poly(vinyl alcohol)/*S. cumini* leaves extract and poly(vinyl alcohol)/chitosan/*S. cumini* leaves extract, adopting a solution-casting method. Films containing cassava starch, laponite, and *S. cumini* fruit anthocyanins manufactured by thermo-compression were used to monitor the meat freshness. Films showed visible changes from purple to yellow color when used to monitor the freshness of round steak stored at −20, 4, and 20 °C [153].

## 5. Summary and Future Perspectives

*S. cumini* contains valuable phytochemicals that are potential drug compounds for the treatment of a wide range of diseases. When incorporated into the diet, some of these compounds could serve as preventative treatments. Additionally, parts of the plant (skin, pulp, roots, leaves, bark) as well as their isolated compounds (quercetin, myricetin, gallic acid, caffeic acid, ellagic acid, delphinidin-3,5-*O*-diglucoside, petunidin-3,5-*O*-diglucoside, malvidin-3,5-*O*-diglucoside) can be used in the food industry with applications in food packaging and as food additives. The present review discussed in detail the health-promoting potential of the various anatomical parts of *S. cumini* and the compounds extractable from those parts. Future studies should be conducted with a view to the isolation and purification of compounds from the different parts of *S. cumini* for treating various ailments. More importantly, clinical investigations are a critical requirement for the discovery of cost-effective drugs that possess a low therapeutic index. Following the aphorism of the “Father of Medicine”, Hippocrates (460–375 BC), “Let food be thy medicine and medicine be thy food”, we should look back to compounds derived from nature with the application of current knowledge and technologies. We should also develop standardized in vitro ‘proof-of-concept’ methods for rigorous investigation of traditional use natural products, which are, unfortunately, often reported with various doses, various purities, and different exposure times, making it difficult to objectively compare and verify their potency and toxicity.

## Figures and Tables

**Table 1:** Content of nutrients in parts of *S. cumini* fruits, seeds, and leaves.

Nutrients	Amount	References
**Fruit**		
Moisture	79.2–85.9%	[3,12,13,14,15,16]
Carbohydrates	7.88–22.4%	[3,12,14,15,16,17]
Proteins	0.65–6.60%	[3,12,13,14,15,16,17,18]
Fats	0.15–1.81%	[3,12,14,15,16,17,18]
Crude fiber	0.22–3.65%	[3,12,14,15,16,17,18]
Ash	0.30–4.50%	[3,13,15,16,17,18]
Calcium	0.02–116.0 mg/100 g	[3,13,14,15,19]
Sodium	3.50–141.7 mg/100 g	[3,14,16]
Potassium	172–1791 mg/100 g	[3,16,19]
Iron	0.10–4.60 mg/100 g	[3,13,14,15,16,19]
Magnesium	9.14–49.8 mg/100 g	[3,12,14,16]
Phosphorus	0.01–18.5 mg/100 g	[13,14,15,19]
Zinc	0.28–2.11 mg/100 g	[3,14,16,19]
Copper	0.07–6.80 mg/100 g	[12,14,16]
Chlorine	8.00 mg/100 g	[12]
Manganese	0.57–1.33 mg/100 g	[16,19]
Chromium	0.35 mg/100 g	[16]
Riboflavin	0.009–0.01 mg/100 g	[13,14,15]
Thiamine	0.008–0.12 mg/100 g	[12,13,14,15,19]
Niacin	0.20–0.30 mg/100 g	[12,13,15,19]
Ascorbic acid	5.70–137 mg/100 g	[3,13,14,16,17,19]
Vitamin A	80 I.U	[12]
Folic acid	3.00 mg/100 g	[12,15]
**Seed**		
Moisture	47.0–52.2%	[3,18]
Carbohydrates	41.0–89.7%	[3,18,20]
Proteins	4.68–6.80%	[3,18]
Fats	0.35–1.28%	[3,18,20]
Ash	2.00–3.13%	[3,18,20]
Calcium	0.41–135 mg/100 g	[3,20]
Magnesium	111.6 mg/100 g	[3]
Potassium	606 mg/100 g	[3]
Phosphorus	0.17%	[20]
Sodium	6.10–43.9 mg/100 g	[3,20]
Iron	4.20 mg/100 g	[3]
Copper	2.13 mg/100 g	[3]
Ascorbic acid	1.84%	[3]
**Leaf**		
Proteins	9.10%	[21]
Fats	4.30%	[21]
Fiber	17.0%	[21]
Ash	6.00%	[21]
Calcium	1.30%	[21]

**Table 2:** Phytochemicals in *S. cumini*.

Plant Part	Phytochemicals	References
**Seeds**	Fatty acids: oleic acid, stearic acid, octadecanal, 1-monolinoleoylglycerol trimethylsilyl ether, *n*-hexadecanoic acid Phenolic acids: gallic acid, ellagic acid Flavonoids: quercetin Phytosterols: *β*-sitoterol Tannins: corilagin, 3,6-hexahydroxy diphenoylglucose, 1-galloylglucose, 3-galloylglucose, 4,6 hexahydroxydiphenoylglucose Others: 2-bromo-cyclohexasiloxane, dodecamethyl, cycloheptasiloxane, tetradecamethyl, pyrazole[4,5-b] imidazole, 1-formyl-3-ethyl-6-beta-d-ribofuranosyl, 3-(octadecyloxy) propyl ester, benzaldehyde	[32,33,34]
**Leaves**	Alkanes: *n*-heptacosane, *n*-nonacosane, *n*-hentriacontane, noctacosanol, *n*-triacontanol, *n*-dotricontanol Terpenoids: betulinic acid, maslinic acid, α-pinene, camphene, globulol, caryophyllene, δ-cadinene, β-eudesmol, β-pinene, γ-cadinene, α-terpineol, camphor, humulene 6,7-epoxide, cubeban-11-ol, α-muurolene, epicubenol, α-copaene, viridiflorene, guanine, β-bourbonene, terpinen-4-ol, endo-borneol, levoverbenone Flavonoids: quercetin, myricetin, myricitrin, flavonol glycosides, myricetin 3-*O*-(4’’-acetyl)-*α* L-rhamnopyranosides Phytosterols: *β*-sitosterol	[31,32,35]
**Stem bark**	Terpenoids: friedelin, friedelan-3-*α*-ol, betulinic acid Phytosterols: *β*-sitosterol, *β*-sitosterol-D-glucoside Phenolic acids: gallic acid, ellagic acid Tannins: gallotannin, ellagitannins Flavonoids: kaempferol, myricetin	[32,33]
**Pulp and Skin**	Anthocyanins: delphinidin-3,5-*O*-digalactoside, delphinidin-3,5-*O-*diglucoside, delphinidin-3-*O*-glucoside, petunidin-3,5-*O*-digalactoside, petunidin-3,5-*O-*diglucoside, petunidin-3-*O*-glucoside, cyanidin-3,5-*O*-digalactoside, cyanidin-3-*O*-glucoside, peonidin-3,5-*O*-digalactoside, peonidin-3,5-*O*-diglucoside, malvidin-3,5-*O*-digalactoside, malvidin-3,5-*O-*diglucoside, malvidin-3-*O*-glucoside Flavonols: myricetin-3-*O*-glucuronide, myricetin-3-*O*-galactoside, myricetin-3-*O*-glucoside, myricetin-3-*O*-rhamnoside, myricetin-3-*O*-pentoside, laricitrin-3-*O*-galactoside, laricitrin-3-*O*-glucoside, syringetin-3-*O*-galactoside, syringetin-3-*O*-glucoside,Flavanonols: DHQ-dihexoside-1, DHQ-dihexoside-2, DHQ-dihexoside-3, MDHQ-dihexoside, MDHQ-dihexoside, DHM-dihexoside-1, DHM-dihexoside-2, DHM-dihexoside-3, DHM-dihexoside-4, DHM-dihexoside-5, DHM-dihexoside-6, MDHM-dihexoside-1, MDHM-dihexoside-2, MDHM-dihexoside-3, MDHM-dihexoside-4, MDHM-dihexoside-5, MDHM-dihexoside-6, DMDHM-dihexoside-1, DMDHM-dihexoside-2, DMDHM-dihexoside-3, liquiritigenin Flavan-3-ols: catechin, epicatechin, gallocatechin, epigallocatechin, epicatechin 3-*O*-gallate, catechin 3-*O*-gallate, epigallocatechin 3-*O*-gallate, gallocatechin 3-*O*-gallate Tannins: galloyl-glucose, 3galloyl-glucose-1, 2galloyl-glucose, 3galloyl-glucose-2, 3galloyl-glucose-3, 3galloyl-glucose-4, 4galloyl-glucose-1, 4galloyl-glucose-2, 5galloyl-glucose-1, 5galloyl-glucose-2, 5galloyl-glucose-3, 6galloyl-glucoside-1, 6galloyl-glucoside-1, castalagin, vescalagin, (2) HHDP-glucose-1, (2) HHDP-glucose-2, G-(2) HHDP-glucose-1, (2) HHDP-glucose-2, (2) G-HHDP-glucose-1, (2) G-HHDP-glucose-2, (2) G-HHDP-glucose-3, (3) G-HHDP-glucose, trisgalloyl-HHDP-glucose-1, trisgalloyl-HHDP-glucose-2 Phenolic acids: quinic acid, gallic acid, chlorogenic acid, caffeic acid, Coumarins: umbelliferon, scopoletin, Terpenoid: rosmanol	[28,32,36,37,38]
**Flowers**	Flavonoids: isoquercetin, quercetin, kaempferol, myricetin, Terpenoid: oleanolic acid, Phenolic acid: ellagic acids	[32]
**Essential oils**	Terpenoids: *α*-terpeneol, myrtenol, eucarvone, muurolol, *α*-myrtenal, 1, 8-cineole, geranyl acetone, *α*-cadinol, pinocarvone	[39]

DHQ, dihydroquercetin; MDHQ, methyl-dihydroquercetin; DHM, dihydromyricetin; MDHM, methyl-dihydrolmyricetin; DMDHM, dimethyl-dihydromyricetin. G, number (n) of Galloyl; HHDP, number (n) of hexahydroxydiphenoyl.

**Table 3:** Anti-inflammatory activity of *S. cumini*.

Plant Part	Extraction Solvent	Species/Assays/Cell Lines	Results	References
**Anti-Inflammatory Activity**				
Fruit	Water	Rats	68.9% inhibition against carrageenan-induced paw edema	[44]
Fruit	Water	Lymphocytes, neutrophils and monocytes	Palliate inflammatory reactions against hepatitis B vaccine	[70]
Fruit	Methanol	Rats	70% inhibition against carrageenan-induced edema	[37]
Fruit	Methanol	Mice	72% inhibition against formaldehyde-induced edema	[37]
Fruit	Methanol	Rats	69% inhibition against PGE2-induced edema	[37]
Fruit	Methanol	Heat-induced hemolysis	67% inhibition against heat-induced hemolysis	[37]
Fruit	Methanol	Serum albumin denaturation	82% inhibition against bovine serum albumin denaturation	[37]
Fruit	Methanol	Egg albumin denaturation	75% inhibition against egg albumin denaturation	[37]
Seed	Methanol	Heat-induced hemolysis	Notable inhibition recorded against heat-induced hemolysis	[75]
Seed	Water	Neutrophils	Significant inhibition of neutrophil chemotaxis	[73]
Seed	Water	Rats	Significant suppression of ectonucleotidase	[74]
Seed	Successive	RAW 264.7	Suppression of pro-inflammatory cytokines (IL-6, IL-10, INF-γ, IL-β, TNF-α)	[77]
Leaf	Ethyl acetate	Rats	Altered C48/80 induced paw edema even at dose of 0.01 mg/kg	[81]
Leaf	Successive	Rats	Significant alteration observed against carrageenan-induced inflammation	[82]
Leaf	Methanol	Rats	Oral administration of 100 and 200 mg/kg exhibited significant anti-inflammatory activity in a dose-dependent manner	[86]
Leaf	Essential Oil	Mice	Significant apoptosis observed among inflammatory cells	[88]
Leaf	Essential Oil	Mice	67% inhibition of eosinophils migration	[89]
Leaf	Water	Mice	Significant inhibition against indomethacin-induced inflammation	[76]
Leaf	Methanol	Rats	75.2% inhibition against carrageenan-induced paw edema	[66]
Stem bark	Ethanol	Rats	40.6% against formaldehyde-induced edema	[48]
Stem bark	Ethanol	Rats	46.0% against PGE2-induced paw edema	[79]
Root	Water	RAW 264.7	Significant reduction of IL-6	[80]

**Table 4:** Anticancer activity of *S. cumini*.

Plant Part	Extraction Solvent	Cell Line	Results	References
Peel	50% aqueous Methanol	SiHa and HeLa	Dose-dependent cell death against both cell lines	[93]
Pulp	Chloroform	PA-1	IC_50_ 27 μg/mL IC_50_ 40 μg/mL	[27]
Pulp	Methanol	OSCC	IC_50_ < 50 μg/mL	[95]
Pulp	Ethanol	Leukemia cells	Direct correlation observed between antioxidant status and anti-leukemia activity	[96]
Pulp	Acidic methanol	HCT-116	Dose-dependent cell death	[94]
Pulp	Methanol	H460	IC_50_ 35.2 µg/mL	[97]
Fruit	Ethanol	HT-29	IC_50_ 267.5 μg/mL	[98]
Seed	Ethanol	A_2_780	IC_50_ 49 μg/mL	[51]
Seed	Ethyl acetate	MCF-7	Dose-dependent cell death	[94]
Seed	Methanol	HCT-116	IC_50_ 1.24 µg/mL	[99]
Leaf	Ethanol	T47D	69% inhibition	[49]
Leaf	Methanol	BM-MSCs	Concentration-dependent cytoprotective activity against H_2_O_2_-treated bone marrow mesenchymal stem cells of rats	[66]
Leaf	Methanol	HCT-116	IC_50_ 1.42 µg/mL	[99]

## Data Availability

Not applicable.

## References

[1] Baliga M.S., Bhat H.P., Baliga B.R.V., Wilson R., Palatty P.L. (2011). Phytochemistry, traditional uses and pharmacology of Eugenia jambolana Lam. (black plum): A review. Food Res. Int..

[2] Baliga M.S., Fernandes S., Thilakchand K.R., D’souza P., Rao S. (2013). Scientific validation of the antidiabetic effects of *Syzygium jambolanum* DC (black plum), a traditional medicinal plant of India. J. Altern. Complement. Med..

[3] Ghosh P., Radha N.R.C., Mishra S., Patel A.S., Kar A. (2017). Physicochemical and nutritional characterization of jamun (*Syzygium cumini*). Curr. Res. Nutr. Food Sci..

[4] Jain A., Katewa S.S., Galav P.K., Sharma P. (2005). Medicinal plant diversity of Sitamata wildlife sanctuary, Rajasthan, India. J. Ethnopharmacol..

[5] Sharma H.K., Chhangte L., Dolui A.K. (2001). Traditional medicinal plants in Mizoram India. Fitoterapia.

[6] Nagaraju G.J., Sarita P., Ramana Murty G.A., Ravi Kumar M., Reddy B.S., Charles M.J. (2006). Estimation of trace elements in some antidiabetic medicinal plants using PIXE technique. Appl. Radiat. Isot..

[7] Ayyanar M., Subash-Babu P. (2012). *Syzygium cumini* (L.) Skeels: A review of its phytochemical constituents and traditional uses. Asian Pac. J. Trop. Biomed..

[8] Ayyanar M., Subash-Babu P., Ignacimuthu S. (2013). *Syzygium cumini* (L.) Skeels., a novel therapeutic agent for diabetes: Folk medicinal and pharmacological evidences. Complement. Ther. Med..

[9] Nadkarni K.M. (1976). Indian Materia Medica.

[10] Chhetri D.R., Parajuli P., Subba G.C. (2005). Antidiabetic plants used by Sikkim and Darjeeling Himalayan tribes, India. J. Ethnopharmacol..

[11] Bhandary M.J., Chandrashekar K.R., Kaveriappa K.M. (1995). Medical ethnobotany of the Siddis of Uttara Kannada district Karnataka India. J. Ethnopharmacol..

[12] Morton J.F. (1987). Fruits of Warm Climates.

[13] Munsell H.E., Williams L.O., Guild L.P., Troescher C.B., Nightingale G., Harris R.S. (1949). Composition of food plants of central America. 1. Honduras. Food Res..

[14] Paul D.K., Shaha R.K. (2004). Citrus fruits in the northern region of Bangladesh. Pakistan J. Biol. Sci..

[15] Radha T., Mathew L. (2007). Fruit Crops.

[16] Shahnawaz M., Sheikh S.A., Nizamani S.M. (2009). Determination of nutritive values of Jamun fruit (*Eugenia jambolana*) products. Pakistan J. Nut..

[17] Gajera H.P., Gevariya S.N., Patel S.V., Golakiya B.A. (2018). Nutritional profile and molecular fingerprints of indigenous black jamun (*Syzygium cumini* L.) landraces. J. Food. Sci. Technol..

[18] Benherlal P.S., Arumughan C. (2007). Chemical composition and in vitro antioxidant studies on *Syzygium cumini* fruit. J. Sci. Food Agric..

[19] Noomrio M.H., Dahot M.U. (1996). Nutritive value of *Eugenia jambosa* fruit. J. Islam. Aced. Sci..

[20] Bhatia I.S., Bajaj K.L. (1975). Chemical constituents of the seeds and bark of *Syzygium cumini*. Planta Med..

[21] Giovannucci E., Rimm E.B., Liu Y., Stampfer M.J., Willett W.C. (2002). A prospective study of tomato products, lycopene, and prostate cancer risk. J. Natl. Cancer Inst..

[22] Wester P.J. (1924). The Food Plants of the Philippines.

[23] Kumar A., Jayach T., Aravindhan P., Deecaraman D., Ilavarasan R., Padmanabhan N. (2009). Neutral components in the leaves and seeds of *Syzygium cumini*. Afr. J. Pharm. Pharmacol..

[24] Daulatabad C.M.J.D., Mirajkar A.M., Hosamani K.M., Mulla G.M.M. (1988). Epoxy and cyclopropenoid fatty acids in *Syzygium cuminii* seed oil. J. Sci. Food Agric..

[25] Hemavathi G.N., Patil S.V., Swamy G.S.K.S.T., Tulsiram K. (2019). Effect of zinc and boron on physical parameters of fruit, seed and quality of Jamun (*Syzygium cumini* Skeels). Int. J. Chem. Stud..

[26] Veigas J.M., Narayan M.S., Laxman P.M., Neelwarne B. (2007). Chemical nature, stability and bioefficacies of anthocyanins from fruit peel of *Syzygium cumini* Skeels. Food Chem..

[27] Li L., Adams L.S., Chen S., Killian C., Ahmed A., Seeram N.P. (2009). Eugenia jambolana Lam. berry extract inhibits growth and induces apoptosis of human breast cancer but not non-tumorigenic breast cells. J. Agric. Food Chem..

[28] de Carvalho Tavares I.M., Lago-Vanzela E.S., Rebello L.P.G., Ramos A.M., Gomez-Alonso S., Garcia-Romero E., Hermosin-Gutierrez I. (2016). Comprehensive study of the phenolic composition of the edible parts of jambolan fruit (*Syzygium cumini* (L.) Skeels). Food. Res. Int..

[29] Franco R.R., Zabisky L.F.R., de Lima Júnior J.P., Alves V.H.M., Justino A.B., Saraiva A.L., Espindola F.S. (2020). Antidiabetic effects of *Syzygium cumini* leaves: A non-hemolytic plant with potential against process of oxidation, glycation, inflammation and digestive enzymes catalysis. J. Ethnopharmacol..

[30] Vijayanand P., Jagan Mohan Rao L., Narasimham P. (2001). Volatile flavour components of jamun fruit (*Syzygium cumini* L.). Flavour. Fragr. J..

[31] Sarma N., Begum T., Pandey S.K., Gogoi R., Munda S., Lal M. (2020). Chemical composition of *Syzygium cumini* (L.) Skeels leaf essential oil with respect to its uses from North East region of India. J. Essent. Oil-Bear. Plants.

[32] Sagrawat H., Mann A., Kharya M. (2006). Pharmacological potential of *Eugenia jamuna*: A Review. Pharm. Mag..

[33] Rastogi R.M., Mehrotra B.N. (1990). Compendium of Indian Medicinal Plants.

[34] Ah A.J., Mahdi J.F., Farooqui M., YH S. (2019). Gas Chromatography-mass spectroscopic analysis of black plum seed (*Syzygium cumini*) extract in hexane. Asian J. Pharm. Clin. Res..

[35] Ranjan A., Jaiswal A., Raja R.B. (2011). Enhancement of *Syzygium cumini* (Indian jamun) active constituents by ultra-violent (UV) irradiation method. Sci. Res. Essays.

[36] Saeed A., Kauser S., Iqbal M. (2018). Nutrient, mineral, antioxidant, and anthocyanin profiles of different cultivars of *Syzygium cumini* (Jamun) at different stages of fruit maturation. Pak. J. Bot..

[37] Qamar M., Akhtar S., Ismail T., Yuan Y., Ahmad N., Tawab A., Ismail A., Barnard R.T., Cooper M.A., Blaskovich M.A. (2021). *Syzygium cumini* (L.), Skeels fruit extracts: In vitro and in vivo anti-inflammatory properties. J. Ethnopharmacol..

[38] Koop B.L., Knapp M.A., Di Luccio M., Pinto V.Z., Tormen L., Valencia G.A., Monteiro A.R. (2021). Bioactive compounds from Jambolan (*Syzygium cumini* (L.)) extract concentrated by ultra-and nanofiltration: A potential natural antioxidant for food. Plant Foods Hum. Nutr..

[39] Shafi P.M., Rosamma M.K., Jamil K., Reddy P.S. (2002). Antibacterial activity of *Syzygium cumini* and *Syzygium travancoricum* leaf essential oils. Fitoterapia.

[40] Faria A.F., Marques M.C., Mercadante A.Z. (2011). Identification of bioactive compounds from jambolão (*Syzygium cumini*) and antioxidant capacity evaluation in different pH conditions. Food Chem..

[41] Warrier P.K., Nambiar V.P.K., Ramankutty C. (1996). Indian Medicinal Plants: A Compendium of 500 Species.

[42] Zhang L.L., Lin Y.M. (2009). Antioxidant tannins from *Syzygium cumini* fruit. Afr. J. Biotechnol..

[43] Modi D.C., Patel J.K., Shah B.N., Nayak B.S. (2010). Anti-inflammatory activity of seeds of *Syzygium cumini* Linn. J. Pharm. Educ. Res..

[44] Aqil F., Jeyabalan J., Munagala R., Singh I.P., Gupta R.C. (2016). Prevention of hormonal breast cancer by dietary jamun. Mol. Nutr. Food Res..

[45] Das S., Sarma G. (2009). Study of the hepatoprotective activity of the ethanolic extract of the pulp of *Eugenia jambolana* (jamun) in albino rats. J. Clin. Diagn. Res..

[46] Syama H.P., Arya A.D., Dhanya R., Nisha P., Sundaresan A., Jacob E., Jayamurthy P. (2017). Quantification of phenolics in *Syzygium cumini* seed and their modulatory role on tertiary butyl-hydrogen peroxide-induced oxidative stress in H9c2 cell lines and key enzymes in cardioprotection. J. Food Sci. Technol..

[47] Arun R., Prakash M.V.D., Abraham S.K., Premkumar K. (2011). Role of *Syzygium cumini* seed extract in the chemoprevention of in vivo genomic damage and oxidative stress. J. Ethnopharmacol..

[48] Muruganandan S., Srinivasan K., Chandra S., Tandan S.K., Lal J., Raviprakash V. (2001). Anti-inflammatory activity of *Syzygium cumini* bark. Fitoterapia.

[49] Artanti N., Maryani F., Dewi R.T., Handayani S., Dewijanti I.D., Meilawati L., Udin L.Z. (2019). In vitro antidiabetic, antioxidant and cytotoxic activities of *Syzygium cumini* fractions from leaves ethanol extract. Indones. J. Cancer Chemoprevention.

[50] Xu J., Liu T., Li Y., Liu W., Ding Z., Ma H., Li L. (2019). Jamun (*Eugenia jambolana* Lam.) Fruit extract prevents obesity by modulating the gut microbiome in high fat diet fed mice. Mol. Nutr. Food Res..

[51] Yadav S.S., Meshram G.A., Shinde D., Patil R.C., Manohar S.M., Upadhye M.V. (2011). Antibacterial and anticancer activity of bioactive fraction of *Syzygium cumini* L. seeds. Hayati J. Biosci..

[52] Suleman M., Khan A., Baqi A., Kakar M.S., Ayub M. (2019). Antioxidants, its role in preventing free radicals and infectious diseases in human body. Pure Appl. Biol..

[53] Banerjee A., Dasgupta N., De B. (2005). In vitro study of antioxidant activity of *Syzygium cumini* fruit. Food Chem..

[54] Maria do Socorro M.R., Alves R.E., de Brito E.S., Pérez-Jiménez J., Saura-Calixto F., Mancini-Filho J. (2010). Bioactive compounds and antioxidant capacities of 18 non-traditional tropical fruits from Brazil. Food Chem..

[55] Reynertson K.A., Yang H., Jiang B., Basile M.J., Kennelly E.J. (2008). Quantitative analysis of antiradical phenolic constituents from fourteen edible Myrtaceae fruits. Food Chem..

[56] Vasi S., Austin A. (2009). Effect of herbal hypoglycemics on oxidative stress and antioxidant status in diabetic rats. Open Diabetes J..

[57] Zahra N., Nadir M., Malik A., Shaukat A., Parveen A., Tariq M. (2019). *In vitro* phytochemical screening and antioxidant activity of jamun (*Eugenia jambolana* Linn) plants parts collected from Lahore, Pakistan. Biochem. Mod. Appl..

[58] Aqil F., Gupta A., Munagala R., Jeyabalan J., Kausar H., Sharma R.J., Gupta R.C. (2012). Antioxidant and antiproliferative activities of anthocyanin/ellagitannin-enriched extracts from *Syzygium cumini* L. (Jamun, the Indian Blackberry). Nutr. Cancer..

[59] Sartaj Ali T.M., Abbasi K.S., Ali A., Hussain A. (2015). Some compositional and biochemical attributes of jaman fruit (*Syzygium cumini* L.) from Potowar region of Pakistan. Res. Pharm..

[60] Singh J.P., Kaur A., Shevkani K., Singh N. (2015). Influence of jambolan (*Syzygium cumini*) and xanthan gum incorporation on the physicochemical, antioxidant and sensory properties of gluten-free eggless rice muffins. Int. J. Food Sci. Technol..

[61] Parmar J., Sharma P., Verma P., Goyal P.K. (2010). Chemopreventive action of *Syzygium cumini* on DMBA-induced skin papillomagenesis in mice. Asian Pac. J. Cancer Prev..

[62] Eshwarappa R.S.B., Iyer R.S., Subbaramaiah S.R., Richard S.A., Dhananjaya B.L. (2014). Antioxidant activity of *Syzygium cumini* leaf gall extracts. BioImpacts: BI.

[63] Mohamed A.A., Ali S.I., El-Baz F.K. (2013). Antioxidant and antibacterial activities of crude extracts and essential oils of *Syzygium cumini* leaves. PLoS ONE.

[64] Ramirez R.O., Roa C.C. (2003). The gastroprotective effect of tannins extracted from duhat (*Syzygium cumini* Skeels) bark on HCl/ethanol induced gastric mucosal injury in Sprague-Dawley rats. Clin. Hemorheol. Microcirc..

[65] Sultana B., Anwar F., Przybylski R. (2007). Antioxidant activity of phenolic components present in barks of *Azadirachta indica, Terminalia arjuna, Acacia nilotica*, and *Eugenia jambolana* Lam. trees. Food Chem..

[66] Ahmed R., Tariq M., Hussain M., Andleeb A., Masoud M.S., Ali I., Hasan A. (2019). Phenolic contents-based assessment of therapeutic potential of *Syzygium cumini* leaves extract. PLoS ONE.

[67] Abdin M., Hamed Y.S., Akhtar H.M.S., Chen D., Chen G., Wan P., Zeng X. (2020). Antioxidant and anti-inflammatory activities of target anthocyanins di-glucosides isolated from *Syzygium cumini* pulp by high speed counter-current chromatography. J. Food Biochem..

[68] de Jesus Soares J., da Silva Rosa A., Motta P.R., Cibin F.W.S., Roehrs R., Denardin E.L.G. (2019). Protective role of *Syzygium cumini* leaf extracts against paraquat-induced oxidative stress in superoxide-dismutase-deficient Saccharomyces cerevisiae strains. Acta Sci. Biol. Sci..

[69] Dos Santos M.M., de Souza Prestes A., de Macedo G.T., Ferreira S.A., Vargas J.L.S., Schüler L.C., de Vargas Barbosa N. (2021). *Syzygium cumini* leaf extract protects macrophages against the oxidized LDL-induced toxicity: A promising atheroprotective effect. Biomed. Pharmacother..

[70] Gupta A., Chaphalkar S.R. (2015). Anti-inflammatory activity of flavonoids from medicinal plants against hepatitis B vaccine antigen on human peripheral blood mononuclear cells. Asian J. Pharm. Clin. Res..

[71] Kumar A., Ilavarasan R., Jayachandran T., Deecaraman M., Kumar R.M., Aravindan P., Krishan M.R.V. (2008). Anti-inflammatory activity of *Syzygium cumini* seed. Afr. J. Biotechnol..

[72] Chaudhuri A.N., Pal S., Gomes A., Bhattacharya S. (1990). Anti-inflammatory and related actions of *Syzygium cumini i* seed extract. Phytother. Res..

[73] Ezekiel U., Heuertz R. (2015). Anti-inflammatory effect of *Syzygium cumini* on chemotaxis of human neutrophils. Int. J. Pharma. Phytochem. Res..

[74] Bitencourt P.E., Cargnelutti L.O., Stein C.S., Lautenchleger R., Ferreira L.M., Sangoi M., Moretto M.B. (2017). Anti-inflammatory action of seed extract and polymeric nanoparticles of *Syzygium cumini* in diabetic rats infected with Candida albicans. J. Appl. Pharm. Sci..

[75] Saha R.K., Zaman N.M., Roy P. (2013). Comparative evaluation of the medicinal activities of methanolic extract of seeds, fruit pulps and fresh juice of *Syzygium cumini* in vitro. J. Coast. Life Med..

[76] Chanudom L., Tangpong J. (2015). Anti-inflammation property of *Syzygium cumini* (L.) Skeels on indomethacin-induced acute gastric ulceration. Gastroenterol. Res. Pract..

[77] Syama H.P., Sithara T., Krishnan S.L., Jayamurthy P. (2018). *Syzygium cumini* seed attenuates LPS induced inflammatory response in murine macrophage cell line RAW264. 7 through NF-κB translocation. J. Funct. Food..

[78] Sagor M.A.T., Mohib M.M., Tabassum N., Ahmed I., Reza H. (2016). Fresh seed supplementation of *Syzygium cumini* attenuated oxidative stress, inflammation, fibrosis, iron overload, hepatic dysfunction and renal injury in acetaminophen induced rats. J. Drug Metab. Toxicol..

[79] Muruganandan S., Pant S., Srinivasan K., Chandra S., Tandan S.K., Lal J., Prakash V.R. (2002). Inhibitory role of *Syzygium cumini* on autacoid-induced inflammation in rats. Indian J. Physiol. Pharmacol..

[80] Mueller M., Janngeon K., Puttipan R., Unger F.M., Viernstein H., Okonogi S. (2015). Anti-inflammatory, antibacterial and antioxidant activities of Thai medicinal plants. Int. J. Pharm. Pharm. Sci..

[81] Lima L.A., Siani A.C., Brito F.A., Sampaio A.L.F., Henriques M.D.G.M.O., Riehl C.A.D.S. (2007). Correlation of anti-inflammatory activity with phenolic content in the leaves of *Syzygium cumini* (L.) Skeels (Myrtaceae). Química Nova.

[82] Jain A., Sharma S., Goyal M., Dubey S., Jain S., Sahu J., Kaushik A. (2010). Anti-inflammatory activity of *Syzygium cumini* leaves. Int. J. Phytomed..

[83] Kumar K.P., Prasad P.D., Rao A.N., Reddy P.D., Abhinay G. (2010). Anti-inflammatory activity of *Eugenia jambolana* in albino rats. Int. J. Pharma Bio Sci..

[84] Ajiboye B.O., Ojo O.A., Akuboh O.S., Abiola O.M., Idowu O., Amuzat A.O. (2018). Anti-hyperglycemic and anti-inflammatory activities of polyphenolic-rich extract of *Syzygium cumini* Linn leaves in alloxan-induced diabetic rats. J. Evid. Based Integr. Med..

[85] Kayser M.S., Nath R., Khatun H., Rashid M.A. (2019). Peripheral analgesic and anti-diarrheal activities of leaf of *Syzygium cumini* (L.) Skeel. Bangladesh J. Pharmacol..

[86] Roy A., Bhattacharya S., Pandey J.N., Biswas M. (2011). Anti-inflammatory activity of *Syzygium cumini* leaf against experimentally induced acute and chronic inflammations in rodents. Altern. Med. Stud..

[87] Patil P., Magar K., Bansode T., Gupta A., Chaphalkar S., Patil A., Sherkhane A. (2018). Exploring anti-inflammatory potential in leaves of Jamun (*Syzygium cumini*). IJSRST.

[88] Machado R.R., Jardim D.F., Souza A.R., Scio E., Fabri R.L., Carpanez A.G., Aarestrup F.M. (2013). The effect of essential oil of *Syzygium cumini* on the development of granulomatous inflammation in mice. Rev. Bras. Farmacogn..

[89] Siani A.C., Souza M.C., Henriques M.G., Ramos M.F. (2013). Anti-inflammatory activity of essential oils from *Syzygium cumini* and Psidium guajava. Pharm. Biol..

[90] Bray F., Ferlay J., Soerjomataram I., Siegel R.L., Torre L.A., Jemal A. (2018). Global cancer statistics 2018: GLOBOCAN estimates of incidence and mortality worldwide for 36 cancers in 185 countries. CA Cancer J. Clin..

[91] Khan M.S., Qais F.A., Ahmad I. (2019). Indian berries and their active compounds: Therapeutic potential in cancer prevention. New Look to Phytomedicine.

[92] Roy A., Jauhari N., Bharadvaja N. (2018). Medicinal plants as a potential source of chemopreventive agents. Anticancer Plants: Natural Products and Biotechnological Implements.

[93] Barh D., Viswanathan G. (2008). *Syzygium cumini* inhibits growth and induces apoptosis in cervical cancer cell lines: A primary study. Ecancermedicalscience.

[94] Ruthurusamy S.K., Dheeba B., Hameed S.S., Palanisamy S. (2015). Anti-cancer and anti-oxidative potential of *Syzygium cumini* against breast cancer cell lines. J. Chem. Pharm. Res..

[95] Ezhilarasan D., Apoorva V.S., Ashok Vardhan N. (2019). *Syzygium cumini* extract induced reactive oxygen species-mediated apoptosis in human oral squamous carcinoma cells. J. Oral Pathol. Med..

[96] Afify A.E.M.M., Fayed S.A., Shalaby E.A., El-Shemy H.A. (2011). *Syzygium cumini* (pomposia) active principles exhibit potent anticancer and antioxidant activities. Afr. J. Pharm. Pharmacol..

[97] Charepalli V., Reddivari L., Vadde R., Walia S., Radhakrishnan S., Vanamala J.K. (2016). *Eugenia jambolana* (Java plum) fruit extract exhibits anti-cancer activity against early stage human HCT-116 colon cancer cells and colon cancer stem cells. Cancers.

[98] Khodavirdipour A., Zarean R., Safaralizadeh R. (2021). Evaluation of the anti-cancer effect of *Syzygium cumini* ethanolic extract on HT-29 colorectal cell line. J. Gastrointest. Cancer.

[99] Eldin Elhawary S.S., Elmotyam A.K.E., Alsayed D.K., Zahran E.M., Fouad M.A., Sleem A.A., Abdelmohsen U.R. (2020). Cytotoxic and anti-diabetic potential, metabolic profiling and insilico studies of *Syzygium cumini* (L.) Skeels belonging to family Myrtaceae. Nat. Prod. Res..

[100] Tripathy G., Pradhan D., Pradhan S., Dasmohapatra T. (2016). Evaluation of plant extracts against lung cancer using H460 cell line. Asian J. Pharm. Clin. Res..

[101] Goyal P.K., Verma P., Sharma P., Parmar J., Agarwal A. (2010). Evaluation of anti-cancer and anti-oxidative potential of *Syzygium cumini* against benzo [a] pyrene (BaP) induced gastric carcinogenesis in mice. Asian Pac. J. Cancer. Prev..

[102] Jagetia G.C., Baliga M.S. (2003). Evaluation of the radioprotective effect of the leaf extract of *Syzygium cumini* (Jamun) in mice exposed to a lethal dose of γ-irradiation. Food/Nahrung.

[103] Jagetia G.C., Shetty P.C., Vidyasagar M.S. (2008). Treatment of mice with leaf extract of jamun (*Syzygium cumini* linn. Skeels) protects against the radiation-induced damage in the intestinal mucosa of mice exposed to different doses of γ-radiation. Pharmacol. Online.

[104] Jagetia G.C., Shetty P.C., Vidyasagar M.S. (2012). Inhibition of radiation-induced DNA damage by jamun, *Syzygium cumini* , in the cultured splenocytes of mice exposed to different doses of γ-radiation. Integr. Cancer Ther..

[105] Jagetia G.C., Shetty P.C. (2016). Augmentation of antioxidant status in the liver of Swiss albino mice treated with Jamun (*Syzygium cumini*, Skeels) extract before whole body exposure to different doses of γ-radiation. J. Adv. Res. Biotechnol..

[106] Jagetia G.C., Baliga M.S., Venkatesh P. (2005). Influence of seed extract of *Syzygium cumini* (jamun) on mice exposed to different doses of γ-radiation. J. Radiat. Res..

[107] Kazmi S.A.J., Riaz A., Akhter N., Khan R.A. (2020). Evaluation of wound healing effects of *Syzygium cumini* and laser treatment in diabetic rats. Pak. J. Pharm. Sci..

[108] Chhikara N., Kaur R., Jaglan S., Sharma P., Gat Y., Panghal A. (2018). Bioactive compounds and pharmacological and food applications of *Syzygium cumini*—A review. Food Funct..

[109] Ebrahimi Y., Hasanvand A., Valibeik A., Ebrahimi F., Abbaszadeh S. (2019). Natural antioxidants and medicinal plants effective on hyperlipidemia. Res. J. Pharm. Technol..

[110] Sharma B., Viswanath G., Salunke R., Roy P. (2008). Effects of flavonoid-rich extract from seeds of *Eugenia jambolana* (L.) on carbohydrate and lipid metabolism in diabetic mice. Food Chem..

[111] Sah A.K., Verma V.K. (2011). *Syzygium cumini* : An overview. J. Chem. Pharm. Res..

[112] Raza A., Malook S., Ali M.U., Akram M.N., Wazir I., Sharif M.N. (2015). Antihypercholesterolemic role of ethanolic extract of jamun (*Syzygium cumini*) fruit and seed in hypyercholesterolemic rats. Am. Eurasian J. Agric. Environ. Sci..

[113] Nahid S., Mazumder K., Rahman Z., Islam S., Rashid M.H., Kerr P.G. (2017). Cardio-and hepato-protective potential of methanolic extract of *Syzygium cumini* (L.) Skeels seeds: A diabetic rat model study. Asian Pac. J. Trop. Biomed..

[114] Nanditha A., Ma R.C., Ramachandran A., Snehalatha C., Chan J.C., Chia K.S., Zimmet P.Z. (2016). Diabetes in Asia and the Pacific: Implications for the global epidemic. Diabetes Care.

[115] Achrekar S., Kaklij G.S., Pote M.S., Kelkar S.M. (1991). Hypoglycemic activity of *Eugenia jambolana* and *Ficus bengalensis*: Mechanism of action. In Vivo (Athens, Greece).

[116] Rekha N., Balaji R., Deecaraman M. (2008). Effect of aqueous extract of *Syzygium cumini* pulp on antioxidant defense system in streptozotocin induced diabetic rats. Iran. J. Pharmacol. Ther..

[117] Farhana R., Swarnomoni D. (2012). Effect of ethanolic extract of seed and pulp of *Eugenia jambolana* (Jamun) on alloxan induced diabetic rats. J. Phytother. Pharmaco. Res..

[118] Sharma S.B., Nasir A., Prabhu K.M., Murthy P.S., Dev G. (2003). Hypoglycaemic and hypolipidemic effect of ethanolic extract of seeds of *Eugenia jambolana* in alloxan-induced diabetic rabbits. J. Ethnopharmacol..

[119] Sridhar S.B., Sheetal U.D., Pai M.R.S.M., Shastri M.S. (2005). Preclinical evaluation of the antidiabetic effect of *Eugenia jambolana* seed powder in streptozotocin-diabetic rats. Braz. J. Med. Biol. Res..

[120] Panda D.K., Ghosh D., Bhat B., Talwar S.K., Jaggi M., Mukherjee R. (2009). Diabetic therapeutic effects of ethyl acetate fraction from the roots of *Musa paradisiaca* and seeds of *Eugenia jambolana* in streptozotocin-induced male diabetic rats. Methods Find. Exp. Clin. Pharmacol..

[121] Kumar E.K., Mastan S.K., Reddy K.R., Reddy G.A., Raghunandan N., Chaitanya G. (2008). Anti-arthritic property of the methanolic extract of *Syzygium cumini* seeds. Int. J. Integr. Biol..

[122] Gohil T., Pathak N., Jivani N., Devmurari V., Patel J. (2010). Treatment with extracts of *Eugenia jambolana* seed and *Aegle marmelos* leaf prevents hyperglycemia and hyperlipidemia in alloxan induced diabetic rats. Afr. J. Pharmacy Pharmacol..

[123] Vikrant V., Grover J.K., Tandon N., Rathi S.S., Gupta N. (2001). Treatment with extracts of *Momordica charantia* and *Eugenia jambolana* prevents hyperglycemia and hyperinsulinemia in fructose fed rats. J. Ethnopharmacol..

[124] Grover J.K., Vats V., Rathi S.S. (2000). Anti-hyperglycemic effect of *Eugenia jambolana* and *Tinospora cordifolia* in experimental diabetes and their effects on key metabolic enzymes involved in carbohydrate metabolism. J. Ethnopharmacol..

[125] Sahana D.A., Shivaprakash G., Baliga R., MR A.P., Ganesh J., Pai M.R.S.M. (2010). Effect of *Eugenia jambolana* on plasma glucose, insulin sensitivity and HDL-C levels: Preliminary results of a randomized clinical trial. J. Pharm. Res..

[126] Cavatão de Freitas T., Oliveira R.J., Mendonça R.J. (2019). Identification of bioactive compounds and analysis of inhibitory potential of the digestive enzymes from *Syzygium* sp. extracts. J. Chem..

[127] Karthic K., Kirthiram K.S., Sadasivam S., Thayumanavan B., Palvannan T. (2008). Identification of amylase inhibitors from *Syzygium cumini* Linn seeds. Indian J. Exp. Biol..

[128] Ponnusamy S., Ravindran R., Zinjarde S., Bhargava S., Ravi Kumar A. (2010). Evaluation of traditional Indian antidiabetic medicinal plants for human pancreatic amylase inhibitory effect in vitro. Evid. Based Complement. Altern. Med..

[129] Chaturvedi A., Bhawani G., Agarwal P.K., Goel S., Singh A., Goel R.K. (2009). Antidiabetic and antiulcer effects of extract of Eugenia jambolana seed in mild diabetic rats: Study on gastric mucosal offensive acid-pepsin secretion. Indian J. Physiol. Pharmacol..

[130] Jonnalagadda A., Maharaja K.K., Kumar N.P. (2013). Combined effect of *Syzygium cumini* seed kernel extract with oral hypoglycemics in diabetes induced increase in susceptability to ulcerogenic stimuli. Diabetes Metab. J..

[131] Sidana S., Singh V.B. (2016). Effect of *Syzygium cumini* (Jamun) seed powder on blood pressure in patients with type 2 diabetes mellitus–a double-blind randomized control trial. Int. J. Sci. Res..

[132] Siddiqui S., Sharma B., Ram G. (2014). Anti-hyperglycemic and anti-hyperlipemia effects of *Syzygium cumini* seed in alloxan induced diabetes mellitus in Swiss albino mice (*Mus musculus*). Med. Aromat. Plants.

[133] Pandey M., Khan A. (2002). Hypoglycaemic effect of defatted seeds and water soluble fibre from the seeds of *Syzygium cumini* (Linn.) skeels in alloxan diabetic rats. NISCAIR-CSIR India.

[134] Kumar A., Ilavarasan R., Deecaraman M., Aravindan P., Padmanabhan N., Krishan M.R.V. (2013). Anti-diabetic activity of *Syzygium cumini* and its isolated compound against streptozotocin-induced diabetic rats. J. Med. Plant Res..

[135] Ravi K., Rajasekaran S., Subramanian S. (2005). Antihyperlipidemic effect of *Eugenia jambolana* seed kernel on streptozotocin-induced diabetes in rats. Food Chem. Toxicol..

[136] Ravi K., Ramachandran B., Subramanian S. (2004). Protective effect of *Eugenia jambolana* seed kernel on tissue antioxidants in streptozotocin-induced diabetic rats. Biol. Pharm. Bull..

[137] Bopp A., De Bona K.S., Bellé L.P., Moresco R.N., Moretto M.B. (2009). *Syzygium cumini* inhibits adenosine deaminase activity and reduces glucose levels in hyperglycemic patients. Fundam. Clin. Pharmacol..

[138] Sharafeldin K., Rizvi M.R. (2015). Effect of traditional plant medicines (*Cinnamomum zeylanicum* and *Syzygium cumini*) on oxidative stress and insulin resistance in streptozotocin-induced diabetic rats. J. Basic Appl. Zool..

[139] Sharma A.K., Bharti S., Kumar R., Krishnamurthy B., Bhatia J., Kumari S., Arya D.S. (2012). *Syzygium cumini* ameliorates insulin resistance and β-cell dysfunction via modulation of PPARγ, dyslipidemia, oxidative stress, and TNF-α in type 2 diabetic rats. J. Pharmacol. Sci..

[140] Chirvan-Nia M.M.P., Ratsimamanga A.R. (1972). Régression de la cataracte et de l’hyperglycémie chez le rat des sables (Psammomys obesus) diabétique ayant reçu un extrait de Eugenia jambolana (Lamarck). Comptes Rendus Hebd. Des Séances De L’académie Des Sciences. Ser. D Sci. Nat..

[141] Mulkalwar S., Kulkarni V., Deshpande T., Bhide H., Patel A., Tilak A.V. (2021). Antihyperglycemic Activity of *Syzygium cumini* (Jamun) in Diabetic Rats. J. Pharma Res. Int..

[142] Bi W.P., Man H.B., Man M.Q. (2014). Efficacy and safety of herbal medicines in treating gastric ulcer: A review. World J. Gastroenterol..

[143] Bhowmik D., Gopinath H., Kumar B.P., Kumar K. (2013). Traditional and medicinal uses of Indian black berry. J. Pharma Phytochem..

[144] de Carvalho Bernardo W.L., Boriollo M.F.G., Tonon C.C., da Silva J.J., Cruz F.M., Martins A.L., Spolidorio D.M.P. (2021). Antimicrobial effects of silver nanoparticles and extracts of *Syzygium cumini* flowers and seeds: Periodontal, cariogenic and opportunistic pathogens. Arch. Oral Biol..

[145] Raman R.K., Santhalakshmy S., John S., Bosco D., Ganguly S. (2020). Phytochemical properties of spray dried jamun juice powder as affected by encapsulating agents. J. Pharmacogn. Phytochem..

[146] Shelke G., Kad V., Yenge G., Desai S., Kakde S. (2020). Utilization of jamun pomace as functional ingredients to enhance the physico-chemical and sensory characteristics of ice cream. J. Food Process. Preserv..

[147] Talukder S., Mendiratta S.K., Kumar R.R. (2020). Jamun fruit (*Syzgium cumini*) skin extract based indicator for monitoring chicken patties quality during storage. J. Food Sci. Technol..

[148] Kapoor S., Ranote P.S., Singh B., Sharma S. (2021). Product characterization and antioxidant potential of rice-based jamun (*Syzygium cumini* L.) powder-supplemented extruded snacks. Int. J. Nutra. Funct. Foods Novel Foods.

[149] Panghal A., Kaur R., Janghu S., Sharma P., Sharma P., Chhikara N. (2019). Nutritional, phytochemical, functional and sensorial attributes of *Syzygium cumini* L. pulp incorporated pasta. Food Chem..

[150] Da Silva Filipini G., Romani V.P., Martins V.G. (2020). Biodegradable and active-intelligent films based on methylcellulose and jambolão (*Syzygium cumini*) skins extract for food packaging. Food Hydrocoll..

[151] Merz B., Capello C., Leandro G.C., Moritz D.E., Monteiro A.R., Valencia G.A. (2020). A novel colorimetric indicator film based on chitosan, polyvinyl alcohol and anthocyanins from jambolan (*Syzygium cumini*) fruit for monitoring shrimp freshness. Int. J. Biol. Macromol..

[152] Kasai D., Chougale R., Masti S., Chalannavar R., Malabadi R.B., Gani R. (2018). Influence of *Syzygium cumini* leaves extract on morphological, thermal, mechanical, and antimicrobial properties of PVA and PVA/chitosan blend films. J. Appl. Polym. Sci..

[153] Gaviria Y.A.R., Palencia N.S.N., Capello C., Trevisol T.C., Monteiro A.R., Valencia G.A. (2021). Nanostructured pH-indicator films based on cassava starch, laponite, and Jambolan (*Syzygium cumini*) fruit manufactured by thermo-compression. Starch-Stärke.

